# A new method for predicting uric acid composition in urinary stones using routine single-energy CT

**DOI:** 10.1007/s00240-017-0994-x

**Published:** 2017-06-28

**Authors:** Mats Lidén

**Affiliations:** Department of Radiology, Faculty of Medicine and Health, Örebro University, 701 85 Örebro, Sweden

**Keywords:** Urinary stone, Kidney stone, Uric acid, Computed tomography, Image analysis, Urolithiasis

## Abstract

Urinary stones composed of uric acid can be treated medically. Prediction of uric acid stone type is, therefore, desirable when a urinary stone is diagnosed with unenhanced CT. The purpose of the present study was to describe single-energy thin slice quantitative CT parameters of urinary stones correlated to chemical stone type and to develop a method to distinguish pure uric acid stones (UA) from other stones (non-UA/Mix). Unenhanced thin slice single-energy CT images of 126 urinary stones (117 patients) with known chemical stone type were retrospectively included in the study. Among the included stones, 22 were UA and 104 were non-UA/Mix. The included CT images and Laplacian filtered images of the stones were quantitatively analyzed using operator-independent methods. A post hoc classification method for pure UA stones was created using a combination of cutoff values for the peak attenuation and peak point Laplacian. The stone types differed in most quantitative image characteristics including mean attenuation (*p* < 0.001), peak attenuation (*p* < 0.001), and peak point Laplacian (*p* < 0.001). The sensitivity for the post hoc-developed peak attenuation—peak point Laplacian method for classifying pure UA stones was 95% [21/22, 95% CI (77–100%)] and the specificity was 99% [103/104, 95% CI (95–100%)]. In conclusion, quantitative image analysis of thin slice routine single-energy CT images is promising for predicting pure UA content in urinary stones, with results comparable to double energy methods.

## Introduction

Urinary stone disease is a common urological disease with a life time risk of approximately 10–15% [1]. Non-contrast-enhanced computed tomography (NECT) has emerged as the primary diagnostic tool for urinary stones with excellent sensitivity and specificity, and the ability to provide valuable information regarding differential diagnosis [2, 3].

The role of NECT is not only to diagnose the urinary stone but also to provide information that guides the patient and the referring clinician in management of the disease. The size and location of a ureteral stone predict the chance of spontaneous passage. The stone characteristics of a renal stone are correlated to stone type and prognosis for treatment success [2, 4–8].

The most common urinary stone types are calcium-based (Ca) stones followed by uric acid stones (UA), and infection-related struvite stones. UA stone composition is of particular interest to predict in intra-renal stones, since these stones can be treated through alkalinization of the urine [2, 9].

During the last decade, several studies have investigated the classification of UA stones vs non-UA stones using dual-energy CT (DECT) [10–16]. However, since the diagnosis of a stone generally is performed using single-energy CT, the DECT requires an additional examination. In contrast, if the stone type can be accurately predicted with single-energy NECT, all information can be reported to the clinician on the time of diagnosis, simplifying the management and avoiding additional radiation dose and costs.

Although it is widely known that the attenuation value of a urinary stone in NECT correlates closely to the stone type, there is an overlap in reported attenuation values, especially for small stones [6, 7, 16–19]. Information of other quantitative characteristics that may improve the classification of urinary stones using single-energy NECT are not present in the literature.

The purpose of the present study was, therefore, to describe single-energy thin slice quantitative CT parameters of urinary stones correlated to chemical stone type and to develop a method to distinguish pure UA stones from non-UA stones.

## Materials and methods

### Patient selection

**Fig. 1:**
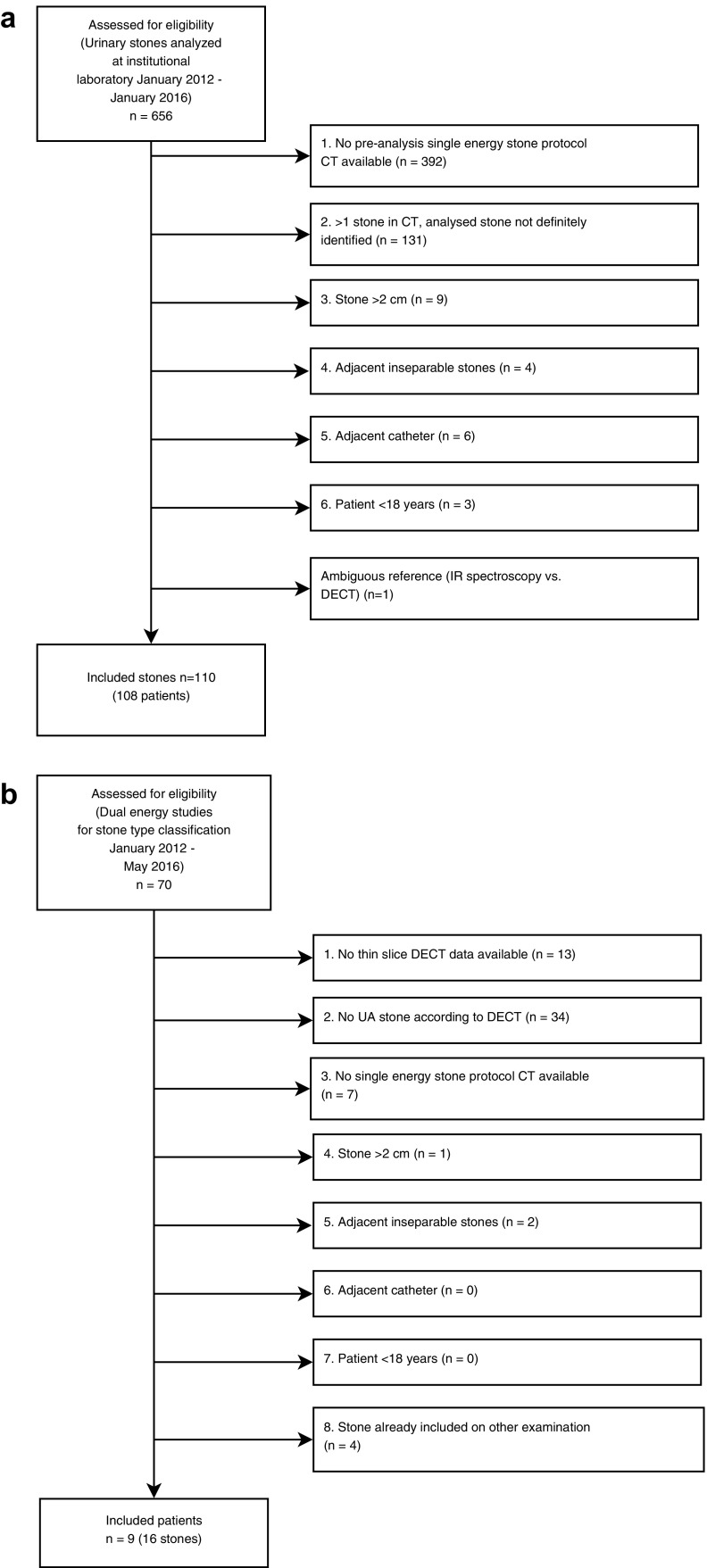
Inclusion process flowcharts. **a** Inclusion based on IR spectroscopy stone analysis. **b** Inclusion of UA stones based on DECT reference

In 108 patients (34 female, 74 male, age 18–85 years) with single-energy NECT examinations performed between January 2012 and January 2016, 110 stones with known compositions were retrospectively included in the study. The inclusion was based on the register of urinary stones analyzed with infrared (IR) spectroscopy at the institutional laboratory between January 2012 and January 2016. Stones were included according to Fig. 1a if the analyzed stone could be identified on pre-analysis thin slice images obtained using the radiology department’s standard NECT protocol for the urinary tract. The median interval between NECT and IR spectroscopy was 42 days (range 7–242 days). 36 stones passed spontaneously, 43 after shock wave lithotripsy, 26 after laser lithotripsy, and 4 stones were surgically removed. The treatment for one stone could not be determined from the radiology information system.

Because of the low number of UA stones included according to Fig. 1a, an additional inclusion of UA stones with dual-energy CT (DECT) as reference was performed. The frequency of UA stones in the DECT cohort was higher since patients referred for DECT generally had clinical suspicion of UA stone disease. In 9 patients (3 female, 6 male) with single-energy NECT examination performed between January 2012 and May 2016, 16 stones with pure UA composition determined by DECT were included according to Fig. 1b, giving a total of 126 included stones. The additional inclusion was based on a list of all DECT scans for stone type classification performed between January 2012 and May 2016. Multiple stones were included in four patients.

The chemical stone composition was determined using Fourier transform IR spectroscopy (Spectrum 100, PerkinElmer, Waltham, MA, USA). The stones were classified as UA, or non-uric acid/mixed (non-UA/Mix) according to Table 1. While the majority of non-UA/Mix stones were calcium-based (Ca), the group also included one struvite stone and two mixed UA/calcium oxalate monohydrate (UA-COM) stones.

**Table 1:** Classification of urinary stones

Non-uric acid/mixed stones (non-UA/Mix) (*n* = 104)
Calcium oxalate monohydrate (*n* = 54)
Calcium oxalate monohydrate with a minor admixture of hydroxyapatite (*n* = 18)
Calcium oxalate monohydrate with traces of hydroxyapatite (*n* = 17)
Mixture of calcium oxalate monohydrate and hydroxyapatite (*n* = 5)
Mixture of calcium oxalate monohydrate and hydroxyapatite with traces of carbonate apatite (*n* = 1)
Hydroxyapatite with a minor admixture of calcium oxalate monohydrate (*n* = 2)
Hydroxyapatite with traces of calcium oxalate monohydrate and carbonate apatite (*n* = 1)
Mixture of hydroxyapatite and carbonate apatite (*n* = 1)
Mixture of hydroxyapatite, carbonate apatite and calcium oxalate monohydrate (*n* = 1)
Mixture of hydroxyapatite and carbonate apatite with traces of magnesium ammonium phosphate (*n* = 1)
Mixture of carbonate apatite and magnesium ammonium phosphate (Struvite) (*n* = 1)
Mixture of uric acid and calcium oxalate monohydrate (UA-COM) (*n* = 2)
Uric acid stones (UA) (*n* = 22)
Uric acid (IR spectroscopy) (*n* = 6)
Uric acid (DECT) (*n* = 16)

The DECT reference scan of the additional UA stones was acquired with a 2 × 128 channel dual-source CT scanner (Siemens Somatom Definition Flash, Siemens Healthcare, Erlangen, Germany). The dual-energy datasets were 100 kV/Sn140 kV, except in one stone where 80 kV/Sn140 kV was used. Color coded images were created using manufacturer default settings of the Kidney stone application in Syngo.Via (14 stones) and in Multi Modality Workplace (2 stones), (Siemens Healthcare, Erlangen, Germany). A stone was considered as a pure UA stone if ≤1 mm DECT high- and low-energy datasets were available and the color coded ≤1 mm thick images were entirely or almost entirely color coded as UA stone. The determination of stone type with DECT was performed before quantitative analysis of the single-energy images.

### Image acquisition

Only 1 mm contiguous axial images performed with the institutional intermediate dose stone NECT protocol (field of view (FoV) 420 mm, in plane pixel size ~0.8 × 0.8 mm, 120 kVp, 70 mAs/slice) were included. The images were acquired with three different CT systems: Philips Brillance, (*n* = 33), 40 channels, filter B, CTDI 4.9 mGy (Philips Medical Systems, Best, The Netherlands); Siemens Somatom Definition AS, (*n* = 15), 64 channels, filter B20f/I30f2, CTDI 5,7 mGy; Siemens Somatom Definition Flash (*n* = 78), filter B20f/I30f3, CTDI 4.7 mGy, except one patient, CTDI 6.1 mGy.

### Image processing

All urinary stones were automatically segmented using a 3D thresholding method where the threshold for each stone was defined as the half value between the peak stone attenuation (maxHU) and the background, which was approximated to 0 HU. Any hole in the thresholded image mask was filled and considered to be part of the stone.

For each segmented stone, histogram statistics were computed (mean attenuation (meanHU), standard deviation (sdHU), kurtosis, and skewness). The stone images were filtered using a scaled 3D Laplacian filter (see “Appendix”) for texture analysis. In the filtered images, the mean Laplacian value (meanLapl) was computed using an image mask corresponding to the segmented stone.

The voxel location corresponding to maxHU was defined as the peak point. The peak point Laplacian (ppLapl) was defined as the voxel in the Laplacian filtered image corresponding to the peak point.

The sphericity and volume of the stone were evaluated using an alpha shape that encompassed all segmented voxel locations (see “Appendix”).

The largest diameter of each stone in the axial slice plane was measured with the caliper tool in the PACS workstation using a bone window setting (L300/W1120) [20].

### Statistical analysis

No predefined hypothesis could be set up prior to image analysis because of the limited availability of quantitative CT analysis data in the literature. The study, therefore, consisted of data exploration for discovering the best combination of quantitative image parameters for stone type prediction. Shapiro–Wilks test showed non-normal distribution for a majority of parameters. Consequently, non-parametric Wilcoxon signed-rank tests between the UA group and the non-UA/Mix group were used. The Spearman correlation coefficient versus maxHU was computed for each parameter.

A post hoc classification method was created using a combination of cutoff values for maxHU and ppLapl. The sensitivity, specificity, accuracy, positive predictive value (PPV), and negative predictive value (NPV) for the peak attenuation—peak point Laplacian method were computed with 95% confidence intervals (CI).

Image analysis and statistics were computed using Matlab R2015b (The MathWorks Inc, Natick, MA, USA). The Regional Research Ethics Board approved the study protocol and waived the informed consent requirement.

## Results

One stone had ambiguous references and was excluded from the analysis according to Fig. 1. The stone was identified as a mixed UA/non-UA stone on a preoperative DECT, but had a fragment collected after ESWL that was IR spectroscopy analyzed as UA. Of the remaining 126 stones, 110 were included after IR spectroscopy and 16 stones were included as UA stones based on DECT, as detailed in Table 1.

The manually estimated largest diameter ± SD was 6.0 ± 2.4 mm and 5.1 ± 2.9 mm for UA and non-UA/Mix stones, respectively.

The quantitative parameters are detailed in Table 2. The stone attenuation, analyzed as maxHU and meanHU, is closely related to the sdHU. The correlation between maxHU, meanHU, and sdHU was almost perfect, indicating that these three variables to the largest part carried the same information.

**Table 2:** Quantitative CT characteristics for the analyzed stone types

	Mean ± 1 SD (range)		Correlation coeff vs. maxHU	Wilcoxon *p* value
	UA (*n* = 22)	Non-UA/Mix (*n* = 104)		
Segmentation-based statistics				
Number of analyzed voxels	188 ± 207 (11–968)	117 ± 188 (6–1166)	0.22	0.001
Mean attenuation, meanHU (HU)	387 ± 98 (240–634)	912 ± 234 (195–1453)	0.99	<0.001
Standard deviation sdHU (HU)	70 ± 19 (47–113)	180 ± 42 (36–284)	0.94	<0.001
Mean stone Laplacian, meanLapl (scaled, HU)	67 ± 29 (34–177)	187 ± 62 (45–328)	0.33	<0.001
Kurtosis	2.6 ± 0.6 (1.7–3.8)	2.3 ± 0.7 (0.3–5.0)	−0.44	0.013
Skewness	0.4 ± 0.4 (−0.5 to 1.2)	0.4 ± 0.4 (−0.7 to 1.4)	−0.38	N/S
Shape statistics^a^				
Sphericity	0.7 ± 0.1 (0.5–0.8)	0.8 ± 0.1^b^ (0.4–0.9)	0.39^b^	0.005^b^
Volume (mm^3^)	89 ± 117 (1–506)	49 ± 97^b^ (0–626)	0.22	0.001
Peak point estimates				
Maximum attenuation, maxHU (HU)	559 ± 139 (365–966)	1275 ± 306 (301–1938)		<0.001
Peak point Laplacian, ppLapl (scaled, HU)	136 ± 46 (62–266)	276 ± 107 (61–507)	0.05	<0.001

^a^The volume and sphericity are based on the alpha shape

^b^In one non-UA/Mix stone, all segmented voxels were located in the same image plane, leading to an alpha shape volume of 0. This stone was not included in the computation of sphericity

The histogram-based statistical properties, kurtosis, and skewness showed moderate correlation with maxHU, while the meanLapl showed low correlation with peak attenuation. The ppLapl was not correlated to maxHU.

Even though the median results were significantly different between the stone types for almost every parameter according to Wilcoxon signed rank test, no single parameter could completely distinguish pure UA stones from the other stone types. The stone attenuation is, as previously known, the major predictor differentiating pure UA stones from non-UA/Mix stones [6, 7, 17–19]. The best combination between the attenuation-based parameter maxHU and a non-attenuation-based parameter was, therefore, sought for.

**Fig. 2:**
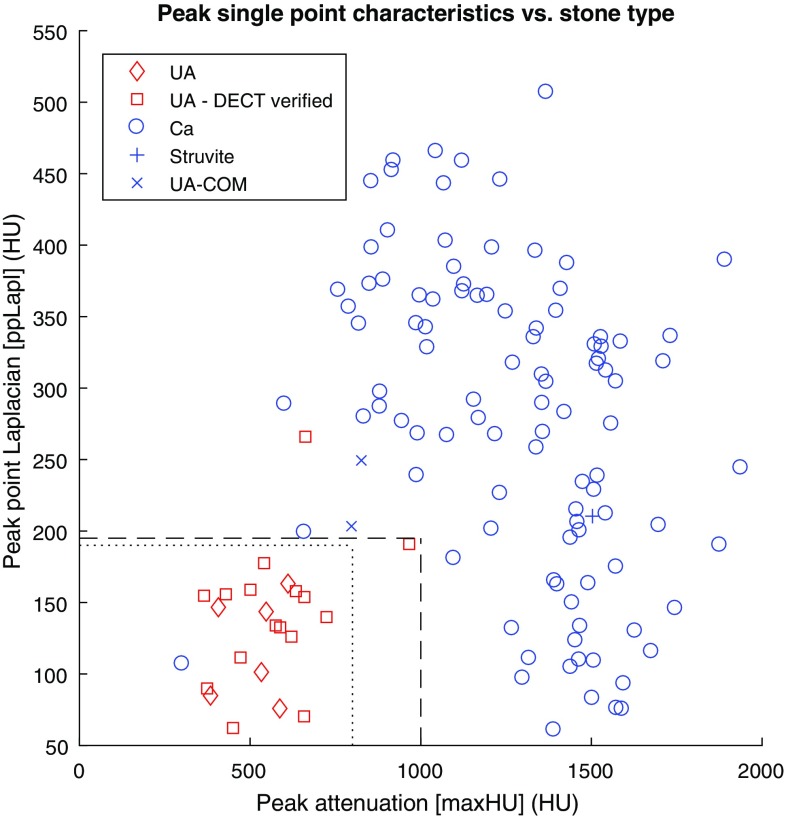
Scatterplot demonstrating the clustering of pure UA stones using the peak attenuation and the peak point Laplacian. The *dashed* (---) *lines* indicate the post hoc-defined cutoff values for pure UA stones 1000 HU/195 HU (maxHU/ppLapl). The *dotted lines* (···) indicate the alternative cutoffs for the major UA cluster (800 HU/190 HU)

Separate scatterplots with maxHU on the *x*-axis were created with volume, meanLapl, kurtosis, skewness, sphericity and ppLapl on the *y*-axis, respectively. The scatterplots were visually evaluated for optimal clustering effects of UA stones vs the non-UA/Mix stones. The visually best discriminant was the combination of peak attenuation and peak point Laplacian, see Fig. 2.

Optimal cutoff values for classification of pure UA stones were created based on visual examination of Fig. 2. A stone was defined as UA stone if maxHU was ≤1000 HU and ppLapl was ≤195 HU. Using this classification, the sensitivity for pure UA stones was 95% [21/22, 95% CI (77–100%)] and the specificity was 99% [103/104, 95% CI (95–100%)]. The PPV was 95% [21/22, 95% CI (77–100%)] and the NPV was 99% [103/104, 95% CI (95–100%)]. The accuracy was 98.4% [124/126, 95% CI (94.4–99.8%)].

With an alternative limit for UA stones (maxHU ≤ 800 HU and ppLapl ≤ 190 HU), the sensitivity was 91% [20/22, 95% CI (71–99%)], the specificity was 99% [103/104, 95% CI (95–100%)] and the accuracy was 97.6% [123/126, 95% CI (93.2–99.5%)].

The optimal cutoff for maxHU as single parameter was 745 HU. With this cutoff the accuracy for classification of pure UA stones was 96.8% [122/126, 95% CI (92.1–99.1%)].

## Discussion

The role of NECT in urinary stone disease is not only to provide the diagnosis but also to provide information that guides the patient and the clinician in the management of the stone [2, 3]. In the present study, it is demonstrated that the peak attenuation of a urinary stone in thin slice single-energy NECT data is an excellent classifier for pure UA stones with an accuracy of 96.8%. With the peak attenuation—peak point Laplacian method that is introduced in this article, the accuracy was even higher, 97.6–98.4% depending on the choice of limits. These results are comparable to the results of DECT [10–12, 14–16].

In almost every analyzed quantitative single-energy parameter, there was a significant difference related to stone type. As previously demonstrated, the stone attenuation is the major predictor of stone type [6, 7, 17–19].

Although the difference in accuracy is small compared to stone type classification based on maxHU alone, a peak attenuation—peak point Laplacian method is suggested based on the clustering demonstrated in Fig. 2, where the combination of maxHU and ppLapl may provide a more specific test than maxHU alone. In contrast to DECT techniques, where the acquisition of two datasets at different kVps are needed for stone classification, the peak attenuation—peak point Laplacian method uses the information that is already present in the routine CT images.

In vivo stone classification using DECT has during the last decade become well established and studies have shown that DECT stone classification can be performed even with low to intermediate dose protocols [12, 15]. However, the availability of DECT is still limited compared to single-energy scanners. The main contribution of the present study is, therefore, not to replace DECT when dual-energy scanning is possible, but rather that it may offer a possibility to avoid the additional radiation and cost of a separate DECT scan when a single-energy NECT has already been performed.

MaxHU and ppLapl are point estimates that have an important advantage, since they are independent of the segmentation parameters. In contrast, the segmentation-based parameters depend not only on the stone but also on the delineation between the urinary stone and the background, which can be achieved differently with or without human interaction [21–25].

**Fig. 3:**
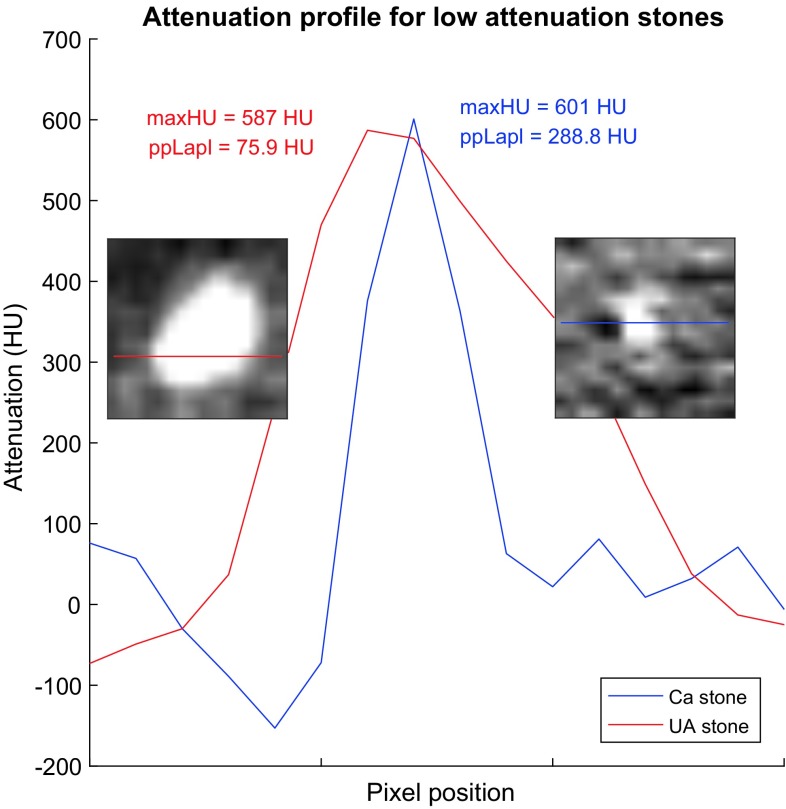
Example of one-dimensional attenuation profiles through a section of a UA and a Ca stone with similar peak attenuation. The corresponding CT images are enclosed. The *different shapes* of the profile *curves* are quantified with the peak point Laplacian value

In digital image processing, the Laplacian is a standard image filter which primarily is used for edge enhancement [26]. In the quantitative context of CT images the discrete Laplacian operator produces quantitative values that have meaningful interpretations. With the scaled 3D Laplacian operator that was used in the study (see “Appendix”), the voxel value in the filtered image corresponds to the difference between the voxel in the unfiltered image and a weighted mean of its 26 neighbors. The ppLapl is thus a measure of the peakedness of the peak point, see Fig. 3.

The difference in ppLapl between UA and Ca stones with low maxHU has a logical interpretation. Low attenuating Ca stones are smaller than UA stones with similar maxHU, leading to a larger peakedness for the Ca stones, quantified by ppLapl. Larger Ca stones show a wide high density peak, resulting in high maxHU and low ppLapl, see Fig. 2.

Several factors contribute to the high accuracy of NECT classification in the present study compared to previous studies. The automated analysis of the stones without the need for freehand ROIs, the consistent use of thin slice images and the use of point estimates that are independent of segmentation parameters are the strengths in the study. Other contributing factors are related to the limitations of the study—the distribution of stone types that was found in the included cohort and the post hoc-defined cutoffs.

Although the number of stones in the present study is larger than in many DECT studies [11, 12, 14–16], further validation of the method, including more UA stones is needed. In the present cohort no cystine stones were present. The differentiation between UA and cystine stones is supposedly challenging because of overlapping attenuation values [18]. As seen in Fig. 2, there is a single outlier UA stone with maxHU 966 HU and ppLapl 191 HU. Further studies will reveal whether the limits for classification of a stone as pure UA may preferably be shifted towards the main UA cluster (for example to maxHU 800 HU and ppLapl 190 HU), leaving an area with maxHU between 800 and 1050 HU and low ppLapl for other stone types such as cystine.

The most important limitation in the present study is that there was no sufficient pre-study quantitative data for setting up a test hypothesis before the study. The sensitivity and specificity is, therefore, likely to be overestimated since the suggested limits were optimized for the existing data.

Struvite stones can often be identified based on morphology [3] and in the present study most struvite stones were excluded based on size during the inclusion process. The two UA/COM stones could not be classified in the present study. Similarly, classification of mixed stones remains a challenge even with DECT [14, 15].

Quantitative image analysis is affected by image parameters including kVp, FoV, slice thickness, and reconstruction algorithm [25]. Even though images from three different CT systems were included in the present study, evaluation and optimization of the stone NECT protocol is necessary for other systems.

## Conclusion

With the limitations of the study in mind, it can be concluded that quantitative image analysis of thin slice single-energy NECT images is promising for predicting pure UA content in urinary stones, with results comparable to DECT. However, further prospective evaluation of the proposed peak attenuation—peak point Laplacian method is needed.

## Appendix

### The Laplacian image filter

The images were filtered using a three-dimensional Laplacian image filter, with the convolution kernel scaled so that the center voxel had coefficient 1, according to Eq. 1. The Laplacian image filter is the discrete approximation of the sum of the second-order derivatives of the intensity values in the image. When properly scaled, the discrete Laplacian image filter produces a weighted mean difference between an intensity value in the unfiltered image and its neighbors. In the present study, a three-dimensional filter was used to include also the neighbors on adjacent slices. The drawback of using the three-dimensional filter is that the CT image data are not completely isotropic, and the noise distribution is different in the z-direction compared to the acquisition plane. Therefore, another option that was not used in the study, would have been to use a two-dimensional Laplacian filter, omitting the information from the adjacent slices

1 $$ {{\text{filter}}\left( { :, : , 1} \right) = - \frac{ 1}{ 9 6} \cdot \left[ {\begin{aligned} 0\\ \quad 3\\\quad 0\\ \end{aligned} \begin{aligned} 3\\\quad 1 0\\ \quad 3\\ \end{aligned} \begin{aligned} 0\\\quad 3\\\quad 0\\ \end{aligned} } \right],\;{\text{filter}}\left( { :, : , 2} \right) = - \frac{ 1}{ 9 6} \cdot \left[ {\begin{aligned} 3\\\quad 1 0\\\quad 3\\ \end{aligned} \begin{aligned} 1 0\\\quad  - 9 6\\\quad  10\\ \end{aligned} \begin{aligned} 3\\\quad  1 0\\\quad  3\\ \end{aligned} } \right],\;{\text{filter}}\left( { :, : , 3} \right) = - \frac{ 1}{ 9 6} \cdot \left[ {\begin{aligned} 0\\\quad  3\\\quad  0\\ \end{aligned} \begin{aligned} 3\\\quad  1 0\\\quad  3\\ \end{aligned} \begin{aligned} 0\\\quad  3\\\quad  0\\ \end{aligned} } \right]} $$

Equation 1: the symmetrical three-dimensional Laplacian image filter used in the study, using Matlab notation.

### Shape analysis

For the shape analysis, the point cloud corresponding to the thresholded three-dimensional-segmented image mask of each urinary stone was translated into a geometrical volume using the alpha shape procedures in the Matlab package (http://www.mathworks.com/help/matlab/ref/alphashape.html). An alpha shape corresponding to a point cloud can be seen as the volume enclosed by a three-dimensional surface consisting of the triangles that are obtained when rolling a sphere of a given radius around the point cloud. The alpha radius of the sphere, that determines the level of detail of the volume, was set to the smallest value that produced an alpha shape that enclosed all segmented voxel center points. From the alpha shape the volume and the surface area of the stone could be computed.

Since the boundary of the alpha shape consists of triangles with coordinates corresponding to the center points of the voxels, there is no 1:1 relationship between the number of voxels in the segmented stone and the volume of the corresponding alpha shape. Especially in small stones, the volume of the alpha shape is considerably lower than the number of voxels multiplied by the voxel size. For example, in a point cloud with eight points forming a perfect cube with a 1 × 1 × 1 mm voxel size, the alpha shape volume is 1 mm^3^, while the number of voxels multiplied by the voxel size is 8 mm^3^.

The sphericity is a measure of the roundness of the stone. The sphericity is defined as the ratio between the surface area of a perfect sphere with the same volume as the lesion and the surface area of the lesion. A perfect sphere consequently has a sphericity value of 1. The sphericity value approaches zero the more a shape deviates from a perfect sphere.
